# The effect and mechanism of selenium supplementation on the proliferation capacity of bovine endometrial epithelial cells exposed to lipopolysaccharide in vitro under high cortisol background

**DOI:** 10.1093/jas/skae021

**Published:** 2024-01-30

**Authors:** Hanqing Li, Junsheng Dong, Luying Cui, Kangjun Liu, Long Guo, Jianji Li, Heng Wang

**Affiliations:** College of Veterinary Medicine, Yangzhou University, Jiangsu Co-innovation Center for Prevention and Control of Important Animal Infectious Diseases and Zoonoses, Yangzhou 225009, China; Joint International Research Laboratory of Agriculture and Agri-Product Safety of the Ministry of Education, Yangzhou 225009, China; International Research Laboratory of Prevention and Control of Important Animal Infectious Diseases and Zoonotic Diseases of Jiangsu Higher Education Institutions, Yangzhou University, Yangzhou 225009, China; College of Veterinary Medicine, Yangzhou University, Jiangsu Co-innovation Center for Prevention and Control of Important Animal Infectious Diseases and Zoonoses, Yangzhou 225009, China; Joint International Research Laboratory of Agriculture and Agri-Product Safety of the Ministry of Education, Yangzhou 225009, China; International Research Laboratory of Prevention and Control of Important Animal Infectious Diseases and Zoonotic Diseases of Jiangsu Higher Education Institutions, Yangzhou University, Yangzhou 225009, China; College of Veterinary Medicine, Yangzhou University, Jiangsu Co-innovation Center for Prevention and Control of Important Animal Infectious Diseases and Zoonoses, Yangzhou 225009, China; Joint International Research Laboratory of Agriculture and Agri-Product Safety of the Ministry of Education, Yangzhou 225009, China; International Research Laboratory of Prevention and Control of Important Animal Infectious Diseases and Zoonotic Diseases of Jiangsu Higher Education Institutions, Yangzhou University, Yangzhou 225009, China; College of Veterinary Medicine, Yangzhou University, Jiangsu Co-innovation Center for Prevention and Control of Important Animal Infectious Diseases and Zoonoses, Yangzhou 225009, China; Joint International Research Laboratory of Agriculture and Agri-Product Safety of the Ministry of Education, Yangzhou 225009, China; International Research Laboratory of Prevention and Control of Important Animal Infectious Diseases and Zoonotic Diseases of Jiangsu Higher Education Institutions, Yangzhou University, Yangzhou 225009, China; College of Veterinary Medicine, Yangzhou University, Jiangsu Co-innovation Center for Prevention and Control of Important Animal Infectious Diseases and Zoonoses, Yangzhou 225009, China; Joint International Research Laboratory of Agriculture and Agri-Product Safety of the Ministry of Education, Yangzhou 225009, China; International Research Laboratory of Prevention and Control of Important Animal Infectious Diseases and Zoonotic Diseases of Jiangsu Higher Education Institutions, Yangzhou University, Yangzhou 225009, China; College of Veterinary Medicine, Yangzhou University, Jiangsu Co-innovation Center for Prevention and Control of Important Animal Infectious Diseases and Zoonoses, Yangzhou 225009, China; Joint International Research Laboratory of Agriculture and Agri-Product Safety of the Ministry of Education, Yangzhou 225009, China; International Research Laboratory of Prevention and Control of Important Animal Infectious Diseases and Zoonotic Diseases of Jiangsu Higher Education Institutions, Yangzhou University, Yangzhou 225009, China; College of Veterinary Medicine, Yangzhou University, Jiangsu Co-innovation Center for Prevention and Control of Important Animal Infectious Diseases and Zoonoses, Yangzhou 225009, China; Joint International Research Laboratory of Agriculture and Agri-Product Safety of the Ministry of Education, Yangzhou 225009, China; International Research Laboratory of Prevention and Control of Important Animal Infectious Diseases and Zoonotic Diseases of Jiangsu Higher Education Institutions, Yangzhou University, Yangzhou 225009, China

**Keywords:** bovine endometrial epithelial cells, cortisol, endometritis, lipopolysaccharide, proliferation, selenium

## Abstract

Bovine endometritis severely inhibits uterine repair and causes considerable economic loss. Besides, parturition-induced high cortisol levels inhibit immune function, reduce cell proliferation, and further inhibit tissue repair. Selenium (Se) is an essential trace element for animals to maintain normal physiological function and has powerful antioxidant functions. This study investigated whether Se supplementation reduces endometrial damage and promotes tissue repair in cows with endometritis under stress and explored the underlying mechanism. Primary bovine endometrial epithelial cells were isolated and purified from healthy cows. The cells were treated with different combinations of lipopolysaccharide (**LPS**), cortisol, and various concentrations of Se. Data showed that LPS stimulation inhibited cell proliferation and increased cell apoptosis. High levels of cortisol further exacerbated these effects. Flow cytometry, scratch wound healing tests, and 5-ethynyl-2’-deoxyuridine (**EdU**) proliferation assays showed that Se supplementation promoted cell cycle progression, cell migration, and cell proliferation in the presence of LPS and cortisol. The quantitative PCR results showed that the expression of related growth factors was increased after Se supplementation. After administering various inhibitors, we further demonstrated that Se supplementation decreased the activity of glycogen synthetase kinase 3β (**GSK-3β**) through the phosphatidylinositol 3-kinase (**PI3K**)/protein kinase B (**AKT**) signaling pathway to reduce the degradation of β-catenin except the Wnt signal to promote cell proliferation. In conclusion, Se supplementation attenuated the cell damage induced by LPS at high cortisol levels and increased cell proliferation to promote uterine repair by elevating the mRNA expression of *TGFB3* and *VEGFA* and activating the PI3K/AKT/GSK-3β/β-catenin signaling pathway.

## Teaser

Selenium supplementation can facilitate bovine endometrial repair.

## Graphical Abstract

**Graphical Abstract:**
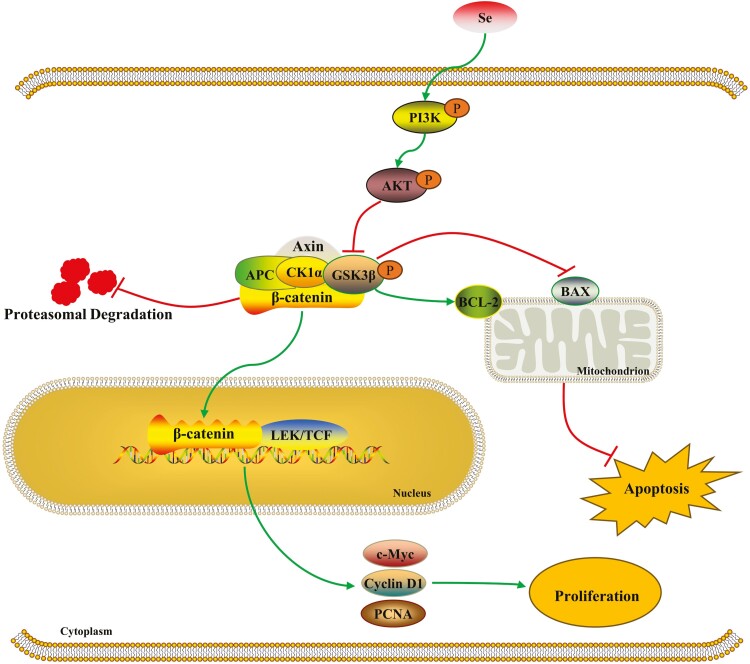

## Introduction

As society and the economy develop, increasing attention is being given to the development of the dairy industry. After parturition, the uterus needs to be quickly repaired to enter the next breeding cycle, thereby generating additional economic value. Usually, as the first line of defense against invading microbial pathogens, bovine endometrial epithelial cells (**BEECs**) will be first regenerated (Wathes et al., 2011). However, 80% to 100% of dairy cows experience bacterial contamination due to the disrupted surface epithelium of the postpartum uterus, and up to 40% eventually develop metritis or endometritis (Paisley et al., 1986; Sheldon et al., 2020). Endometritis delays uterine involution and affects subsequent reproductive performance in bovines, resulting in significant economic losses (Esslemont and Peeler, 1993; Mohammed et al., 2019). Escherichia *coli* is often the infectious agent in the early stages of intrauterine infection and favors the development of uterine infections caused by other bacteria, such as *Trueperella pyogenes* leading to chronic uterine disease (Dohmen et al., 2000). Therefore, preventing *E. coli* infection will prevent the adverse effects associated with lipopolysaccharide (**LPS**) and other bacterial infections during the later postpartum period. For these reasons, *E. coli* was chosen for the construction of a cell model, and LPS was widely used in vitro endometritis models (Zhao et al., 2018; Cui et al., 2021).

Cows usually exhibit increased cortisol (**COR**) levels during the perinatal period due to various stress factors such as parturition, lactation, or improper manual manipulation (Smith et al., 1973; Nagel et al., 2019). High levels of COR due to parturition cause immunosuppression and reduce the ability of the uterus to clear bacterial infections, increasing the severity of uterine infections (Zoldan et al., 2014). In addition, the stress-induced release of COR impairs wound healing (Pombeiro et al., 2022). A previous study revealed that high levels of COR reduce the proliferation of BEECs through the Wnt/β-catenin and phosphatidylinositol 3-kinase (**PI3K**)/protein kinase B (**AKT**) pathways (Dong et al., 2019). Thus, high levels of COR in postpartum cows may affect uterine repair.

Selenium (**Se**) is a vital trace element for animal health and performance. However, Se deficiency is common in cows worldwide (Knowles and Grace, 2014; Müller et al., 2014). During the perinatal period, a negative energy balance often occurs in dairy cows, leading to a decrease in Se content due to the decreases in dry matter intake at the beginning of lactation and near parturition (Ingvartsen and Moyes, 2015). Se deficiency seriously impacts the cow breeding industry because of its deleterious effects on dairy cows’ milk quality and reproductive function (Wang et al., 2022). Se supplementation in cows has been shown to reduce the incidence of endometritis, placental retention, and mastitis (Harrison et al., 1984; Allison and Laven, 2000). To maximize the economic value produced by a cow, the postpartum uterus needs to be repaired as soon as possible to enter the next breeding cycle. The process of endometrial repair is similar to that of wound healing. Se can be mobilized to the injury site to activate early wound healing mechanisms (Mirastschijski et al., 2013). Se supplementation has been shown to reduce the time of bovine uterine involution (Harrison et al., 1986). In goats, Se supplementation can increase the proliferation and decrease the apoptosis of endometrial cells to promote endometrial tissue repair (Li et al., 2023a). In cows, Se supplementation reduces the oxidative stress-induced apoptosis of bovine mammary epithelial cells and increases cell proliferation (Miranda et al., 2011). Therefore, we hypothesized that Se supplementation might regulate the proliferation and apoptosis of bovine endometrial cells to promote uterine repair in cows.

The PI3K/AKT and Wnt/β-catenin signaling pathways have been shown to be closely related to cell proliferation (Hennessy et al., 2005; Wang et al., 2023). When both signaling pathways are activated, BEECs proliferate to promote endometrial repair (Dong et al., 2019).β-catenin is the central signaling protein of the Wnt/β-catenin signaling pathway. Under resting conditions, it is located in the cytoplasm and is regulated by the activity of a destruction complex comprising adenomatous polyposis coli, casein kinase 1α (**CK1α**), the scaffolding protein Axin and glycogen synthetase kinase 3β (**GSK-3β**). Then it is modified by ubiquitin and degraded through proteasomes (Amit et al., 2002). When the PI3K/AKT signaling pathway is activated, the activity of the anti-apoptotic protein B-cell lymphoma 2 (**BCL2**) is inhibited, and the expression of the pro-apoptosis protein BCL2-associated X protein (**BAX**) is upregulated (Kim et al., 2008). Besides, activated AKT can phosphorylate Ser9 in GSK-3β, thereby inactivating GSK-3β (Kim et al., 2013). After that, the degradation complex dissociates, resulting in the accumulation of β-catenin protein in the cytoplasm. Next, the accumulated cytoplasmic β-catenin enters the nucleus, which is a vital step in activating the Wnt signaling pathway. Nuclear β-catenin binds to T-cell factor/lymphoid enhancing factor (**TCF**/**LEF**) to activate oncogenes such as Cyclin D1 and c-Myc (Huber et al., 1996; He et al., 1998; Tetsu and McCormick, 1999). Therefore, GSK-3β is at the intersection of the PI3K/AKT and Wnt/β-catenin signaling pathways. Se can affect the proliferation of cancer cells by regulating the degradation of β-catenin through GSK-3β, but this effect is related to the cell type (Saifo et al., 2010).

At present, studies on whether Se supplementation promotes endometrial repair in dairy cows are rare. Accordingly, in this study, LPS and COR were used to construct a cell model to investigate whether Se supplementation can attenuate cell damage and increase cell proliferation capability to promote uterine repair and clarify the possible underlying mechanism.

## Materials and Methods

All animal care, surgical, and sample collection procedures were approved by the Animal Care and Use Committee of Yangzhou University (approval code: 202212116).

### Isolation and culture of BEECs

Primary BEECs were isolated and cultured as described in a previous article (Dong et al., 2019). In brief, healthy uteri from pre-estrus cows (ovarian stage I) free of microbial infections or genital diseases were collected from a local abattoir. The stage of the reproductive cycle was determined according to a previous article (Ireland et al., 1980). The uterine horns were cut into 3- to 4-cm-long segments, and the endometrium was fully exposed on a clean bench. Then, the uterine tissue was digested with 1 mg/mL 0.1% protease from Streptomyces griseus (P5147, Sigma, USA), 200 μg/mL streptomycin, and 200 units/mL penicillin diluted in DMEM/F12 (D8900, Sigma). After 12 to 18 h of incubation at 4 °C, the endometrium was gently scraped. The scraped endometrium was then washed in phosphate-buffered saline (**PBS**) and centrifuged in PBS at 100 × *g* for 5 min. This step was repeated 3 times, after which the cell suspension was collected. Finally, the cells were cultured in DMEM/F12 medium supplemented with 15% fetal bovine serum (Gibco, USA), 100 units/mL penicillin, and 100 μg/mL streptomycin. The cells were cultured at 37 °C with 5% CO_2_ and 95% sterile air. The medium was changed every 1 to 2 d. The purification of BEECs was confirmed to be greater than 99% by immunohistochemical detection of CK-18.

### Experimental design

On the basis of data from our laboratory (Dong et al., 2018; Cui et al., 2021, 2023), the following concentrations were used: 1, 2, or 4 μM Se (S5261, Sigma), 30 ng/mL COR (H0888, Sigma), and 10 ug/mL LPS (L6529, Sigma). All these reagents were prepared as stock solutions according to the instructions and diluted in DMEM/F12 when used. The BEECs in the experimental groups were pretreated with DMEM-F12 containing Se (1, 2, or 4 μM) for 12 h and then challenged with LPS and COR for 24 h. Six parallel groups were prepared: the control group, the LPS group, the LPS + COR group, and the LPS + COR + Se (1, 2, or 4 µM) groups.

### Cell cycle analysis

The BEECs were detached by gentle trypsinization after treatment. Then, the cells were washed with cold PBS and fixed with ice-cold 70% ethanol for 24 h at 4 °C. After that, the fixed cells were washed twice with cold PBS and stained with propidium iodide and RNase A from a cell cycle and apoptosis analysis kit (C1052, Beyotime, China) in the dark at 37 °C for 30 min in accordance with the manufacturer’s protocol. The cell cycle distribution was determined immediately by flow cytometry (LSRFortessa, BD Biosciences, USA). The data were analyzed using FlowJo software 7.6 (BD Biosciences).

### Cell migration assay

A scratch wound healing assay was used to measure cell migration according to a previously described method (Cui et al., 2021). In brief, BEECs were seeded on a six-well plate, grown to 90% confluence, and then pretreated with various concentrations of Se for 12 h. A scratch was made in the cell monolayer using a 200 μL pipette tip, after which the cells were and then washed with PBS to remove cell debris. Finally, photos of the migrated cells after treatment were taken under an inverted microscope at 100× magnification and analyzed by ImageJ software (Media Cybernetics, Rockville, USA).

### EdU proliferation assay

The BEECs proliferation assay was conducted using a BeyoClick EdU Cell Proliferation Kit with Alexa Fluor 488 (C0071S, Beyotime) according to the manufacturer’s instructions. After treatment, BEECs were labeled with 10 µM EdU for 3 h at 37 °C, fixed with 4% paraformaldehyde for 15 min, and permeabilized with 0.4% Triton X-100 (ST797, Beyotime) for 15 min at 37 °C. After being washed three times with PBS containing 3% BSA, the BEECs were incubated with the Click Reaction Mixture for 30 min and then incubated with Hoechst 33342 for 15 min at 25 °C in the dark. Finally, fluorescence images were acquired by a fluorescence microscope (Leica TCS SP8, Leica Corporation, Germany).

### RNA extraction and quantitative real-time polymerase chain reaction (qPCR)

Total RNA was extracted using the TRIzol reagent (ET111, TRAN, China) and quantified using a Nanodrop 2000 spectrophotometer (Thermo, USA). The OD260 nm/OD280 nm ratio of each sample was determined to be between 1.8 and 2.1. Reverse transcription was conducted with a HiScript II 1st Strand cDNA Synthesis Kit (R211-1, Vazyme, China) according to the manufacturer’s protocol. As described in a previous report (Cui et al., 2021), the primer sequences listed in Table 1 were purified and sequenced by TsingKe Biotech, China. All the sequence results were analyzed using BLAST and compared to the GenBank database. A single product was amplified with each primer pair. Then, qPCR was carried out with a 7500 real-time PCR system (Applied Biosystems, Life Technologies, Corp., USA) and ChamQ SYBR qPCR Master Mix (Q311, Vazyme). The qPCR mixture included was 10 μL of ChamQ SYBR qPCR Master Mix, 0.4 μL of each primer (10 μM), 2 μL of cDNA template, and 7.4 μL of diethylpyrocarbonate-treated water in a final volume of 20 μL per reaction. The reaction cycles for all genes were 95 °C for 30 s, 40 cycles at 95 °C for 10 s, and 60 °C for 30 s. All experiments were repeated in triplicate independently. The products were quantified using the 2^-△△^^Ct^ method and target gene expression was normalized to that of *ACTB*.

**Table 1.** Primer sequences for real-time PCR amplification

Gene	Primer	Sequence (5’ → 3’)	Product (bp)	Accession number
*TGFB1*	Forward	CGAGCCCTGGACACCAACTA	137	NM_001166068.1
	Reverse	AGGCAGAAATTGGCGTGGTA		
*TGFB3*	Forward	CTGTGCGTGAATGGCTCTTG	153	NM_001101183.1
	Reverse	CATCATCGCTGTCCACACCT		
*VEGFA*	Forward	GACCCTGGTGGACATCTTCC	127	NM_001316992.1
	Reverse	CACACAGGGCACACACTCC		
*CCN2*	Forward	AGCTGACCTGGAGGAGAACA	139	NM_174030.2
	Reverse	GTCTGTGCACACTCCGCAGA		
*CTNNB1*	Forward	CCAAGTGGGTGGCATAGAGG	274	NM_001076141.1
	Reverse	CTGCTCACGCAAAGGTGCA		
*ACTB*	Forward	CATCACCATCGGCAATGAGC	156	NM_173979.3
	Reverse	AGCACCGTGTTGGCGTAGAG		

### Protein extraction and Western blotting

Total protein was obtained by homogenizing cells in RIPA Lysate Buffer (P0013B, Beyotime) according to the manufacturer’s instructions. Next, the BCA Protein Assay Kit (Beyotime Biotech, Nantong, China) was used to determine protein concentration. Equal amounts of protein (30 μg) were separated via 10% sodium dodecyl sulfate–polyacrylamide gel electrophoresis (SDS-PAGE, Solarbio, Beijing, China), transferred onto polyvinylidene difluoride membranes (Millipore, Bedford, MA, USA) and incubated with 5% TBST (50 mmol/L Tris, pH 7.6, 150 mmol/L NaCl, and 0.1% Tween 20) containing 5% nonfat milk for 2 h at 25 °C. After washing three times in TBST, the membranes were cut prior to hybridization and incubated overnight with primary antibodies at 4 °C. Primary antibodies against proliferating cell nuclear antigen (**PCNA**; 1/1000, # 10205-2-AP) and BAX (1/5000, # 50599-2-Ig) were purchased from Proteintech Group (Wuhan, China); GSK-3β (1/10000, # ab75814), p-GSK-3β (1/5000, # ab32391), and β-catenin (1/10000, # ab32572) were purchased from Abcam (Shanghai, China); PI3K (1/1000, # 4292), p-PI3K (1/1000, # 4228), AKT (1/1000, # 4691), p-AKT (1/2000, # 4060), Cyclin D1 (1/1000, # 2978), c-Myc (1/1000, # 5605), and β-actin (1/1000, # 4970) were purchased from Cell Signaling Technology (Danvers, MA, USA), and BCL2 (1/1000, # sc-7382) was purchased from Santa (Nanjing, China). Then, the membranes were washed and incubated with the second antibody of HRP* Goat Anti Rabbit IgG (H + L) (RS0002, Immunoway, USA) or Anti-IgG (H + L chain) (Mouse) pAb-HRP (# 330, Marine Biological Laboratory, Japan) for 1 h at 25 °C. After being washed three times in TBST, the immunoreactive bands were visualized by enhanced chemiluminescence (Millipore, Shanghai, China) and imaged using a ChemiScope5300Pro CCD camera (Clinx Science Instruments, Shanghai, China). The experiments were repeated in triplicate for each group, and the data were analyzed using ImageJ software.

### Immunofluorescence staining

The BEECs were seeded on coverslips in 24-well cell culture plates. After treatment, the BEECs were fixed with 4% paraformaldehyde (BL539A, Biosharp) for 15 min at 25 °C, followed by permeabilization with 0.4% Triton X-100 (ST797, Beyotime) for 15 min at 37 °C. After blocking with 5% bovine serum albumin (**BSA**) for 2 h at 25 °C, the cells were incubated at 4 °C overnight with anti-β-catenin (diluted in 5% BSA). Then, the coverslips were washed with PBS and incubated with a FITC-conjugated secondary antibody (A0423, Beyotime) for 1 h at 37 °C. Finally, the cells were dyed with DAPI Staining Solution (C1005, Beyotime) for 15 min at 37 °C in the dark and examined using a fluorescence microscope (Leica TCS SP8, Leica Corporation, Germany).

### Statistical analysis

Three independent replicates of the experiments were performed. The results were analyzed using IBM SPSS Statistics 21.0 (IBM) and expressed as the mean ± standard error of the mean (SEM). Data analyses were performed statistically with IBM SPSS Statistics 21.0 (IBM, USA) by one-way analysis of variance (ANOVA). A *P* value of less than 0.05 was considered a significant difference statistically.

## Results

### Se promoted LPS-inhibited cell cycle progression in BEECs at high COR levels

**Figure 1.**
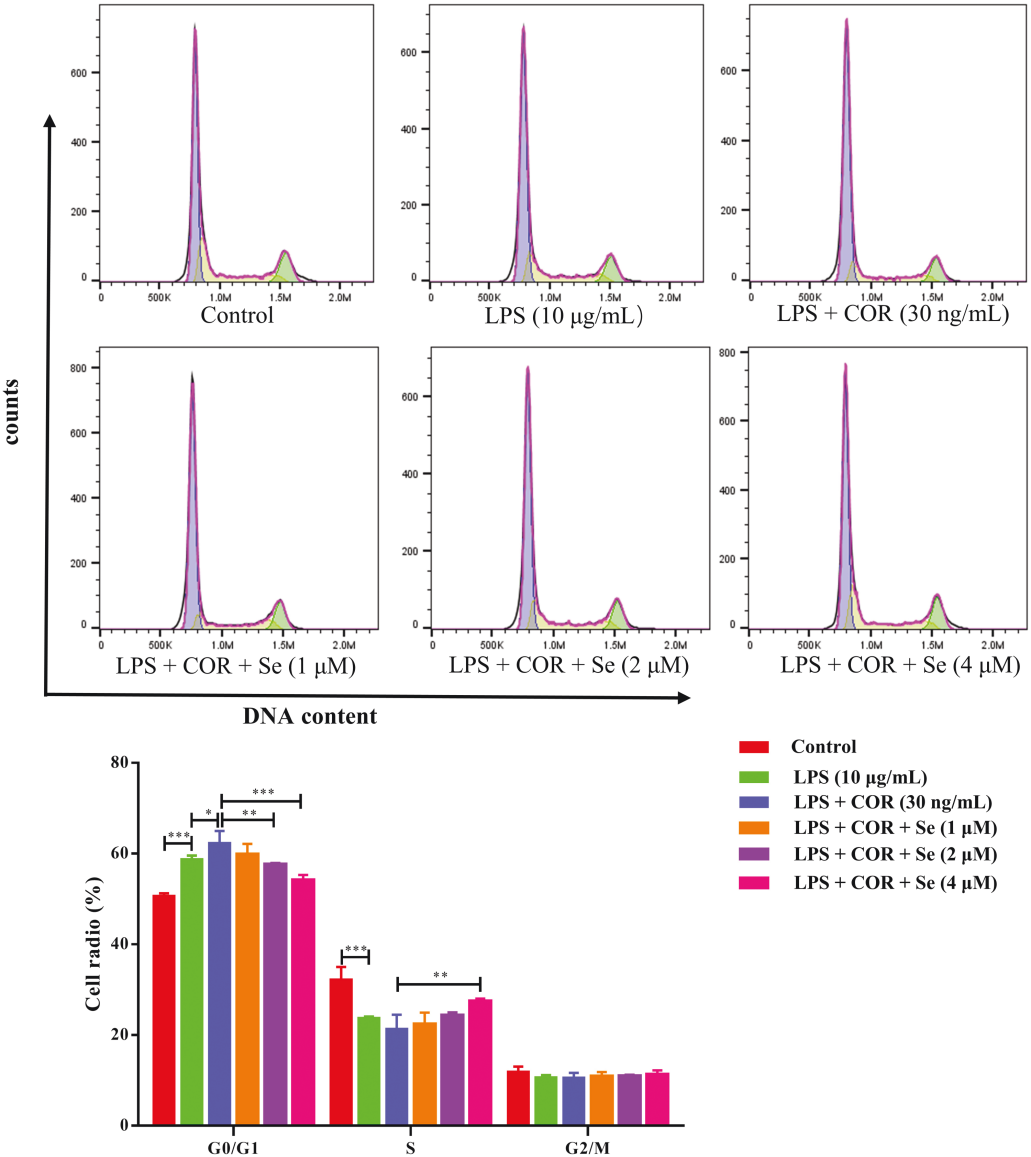
The effect of Se on the LPS-inhibited cell cycle distribution of the BEECs at high COR levels. The cell cycle distribution was detected by flow cytometry after treated with LPS or co-treated with LPS and COR or co-treated with LPS, COR, and Se (1, 2, and 4 μM). *, *P* < 0.05, **, *P* < 0.01, and ***, *P* < 0.001. All data were presented as the means ± SEM (*n* = 3).

As shown in Figure 1, after treatment with 10 μg/mL LPS for 24 h, the number of cells in the G0/G1 phase increased (*P* < 0.001), whereas the number of cells in the G2 phase decreased (*P* < 0.01). Compared with the LPS group, the number of cells in the co-treatment groups with LPS and COR further increased (*P* < 0.001) in the G0/G1 phase, indicating that the cell cycle was arrested in the G0/G1 phase. Compared with the LPS + COR group, the LPS + COR + Se groups had fewer cells in the G0/G1 phase (*P* < 0.01 or 0.001), and more cells in the G2 phase (*P* < 0.01). These results indicated that Se supplementation could promote cell cycle progression in the presence of LPS and COR.

### Se mitigated BEECs impairment and promoted proliferation at high COR levels

**Figure 2.**
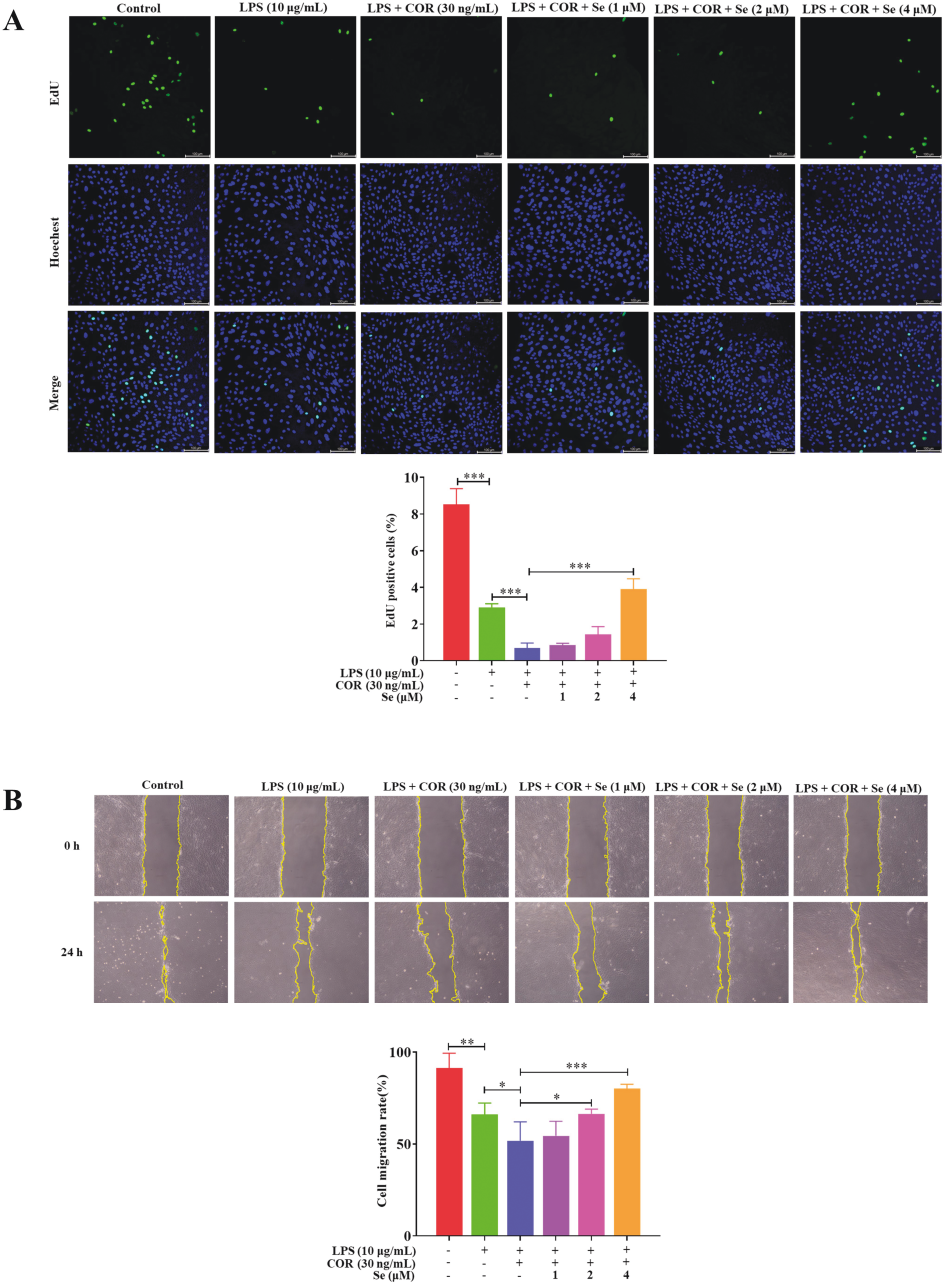
Se enhanced LPS-inhibited migration and proliferation of BEECs at high COR levels. (A) The effect of Se on the cell migration rate of the BEECs by using the scratch wound healing assay. The cell migration rate = (scratch width at 0 h − scratch width at 24 h)/scratch width at 0 h × 100%. The cells were observed under light microscopy at 100× magnification. (B) EdU assay of the cell proliferation ability in BEECs. (A and B) The cells were treated with LPS or co-treated with LPS and COR or co-treated with LPS, COR, and Se (1, 2, and 4 μM). *, *P* < 0.05, **, *P* < 0.01, and ***, *P* < 0.001. The scale bar = 100 μm. All data were presented as the means ± SEM (*n* = 3).

As shown in Figure 2A, the cell migration rate in the LPS group at 24 h was decreased (*P* < 0.01) than that in the control group. Compared with that in the LPS group, the cell migration rate was further reduced (*P* < 0.05) in the LPS + COR group. Compared with that in the LPS + COR group, the cell migration rate was positively correlated with the Se concentration and was increased (*P* < 0.01 or 0.001) at 2 and 4 μM Se. In addition, the results (Figure 2B) showed that the proportion of EdU-positive cells was lower in the LPS group than in the control group and further decreased after the addition of COR. The proportion of EdU-positive cells showed a dose-dependent relationship with the Se concentration and reached its highest value (*P* < 0.001) at 4 μM Se compared with that in the LPS + COR group. These results implied that Se mitigated BEECs impairment and promoted proliferation at high COR levels.

### Se increased the LPS-inhibited secretion of cell-associated growth factors in BEECs at high COR levels

**Figure 3.**
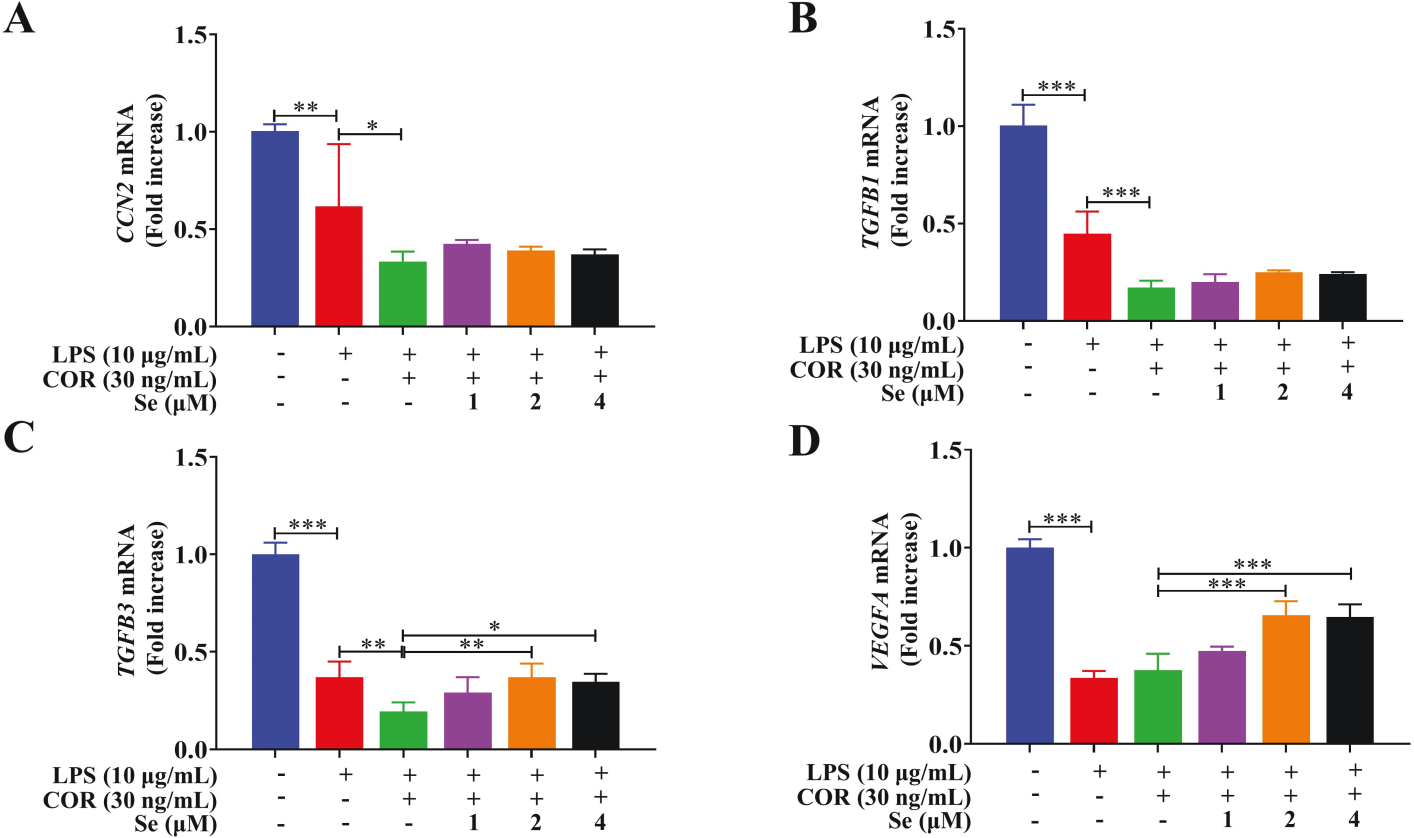
Effects of Se on the gene expressions of CCN2 (A), TGFB1 (B), TGFB3 (C), and VEGFA (D) in BEECs. The cells were treated with LPS or co-treated with LPS and COR or co-treated with LPS, COR, and Se (1, 2, and 4 μM). *, *P* < 0.05, **, *P* < 0.01, and ***, *P* < 0.001. All data were presented as the means ± SEM (*n* = 3).

As shown in Figure 3, LPS treatment decreased (*P* < 0.001) the gene expression of *CCN2*, *TGFB1*, *TGFB3*, and *VEGFA* in BEECs compared with that in the control group. Compared with those in the LPS group, the gene expression levels of *CCN2*, *TGFB1*, and *TGFB3* in the co-treatment group with LPS and COR were further decreased (*P* < 0.05, 0.01 or 0.001). Compared with that in the LPS + COR group, Se treatment increased (*P* < 0.05, 0.01, or 0.001) *TGFB3 and VEGFA* gene expression.

### Se activated the LPS-inhibited PI3K/AKT and Wnt/β-catenin signaling pathways in BEECs at high COR levels

**Figure 4.**
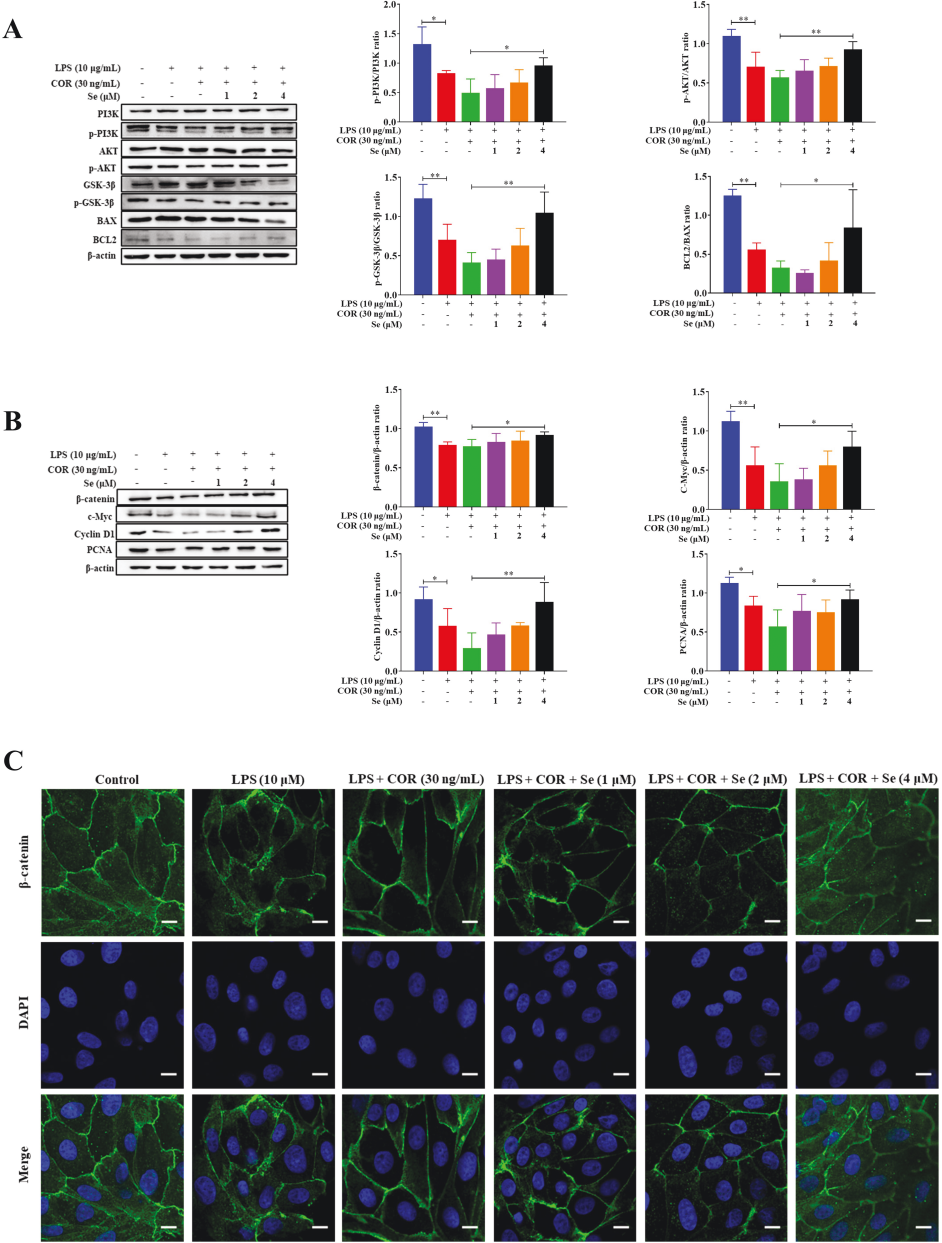
Effects of Se on the PI3K/AKT (A) and Wnt/β-catenin (B) signaling pathways in BEECs. The phosphorylation levels the protein levels were detected by Western blotting. (C) The effect of Se on the nuclear-transport of β-catenin in BEECs. The β-catenin levels were evaluated by confocal microscopy. The scale bar = 10 μm. (A, B, and C) The cells were treated with LPS or co-treated with LPS and COR or co-treated with LPS, COR and Se (1, 2 and 4 μM). *, *P* < 0.05, **, *P* < 0.01, and ***, *P* < 0.001. All data were presented as the means ± SEM (*n* = 3).

The results in Figure 4 showed that the phosphorylation levels of PI3K, AKT, and GSK-3β, the protein levels of β-catenin, c-Myc, Cyclin D1, and PCNA, and the protein ratio of BCL2/BAX in the LPS group were decreased (*P* < 0.05, 0.01. or 0.001) than those in the control group. These indexes increased in a dose-dependent manner with increasing Se concentration and reached their highest values at 4 μM Se, which showed an increase (*P* < 0.05 or 0.01) compared with the LPS + COR group. Besides, Se increased the distribution of β-catenin in the nuclei of BEECs in the LPS + COR + Se groups compared with that in the LPS + COR group. Taken together, these results indicated that Se could activate the LPS-inhibited PI3K/AKT and Wnt/β-catenin signaling pathways to promote BEECs proliferation at high COR levels.

### Se inhibited the LPS-induced activity of GSK-3β and accelerated the degradation of β-catenin protein through ubiquitin–proteasome pathway at high COR levels

**Figure 5.**
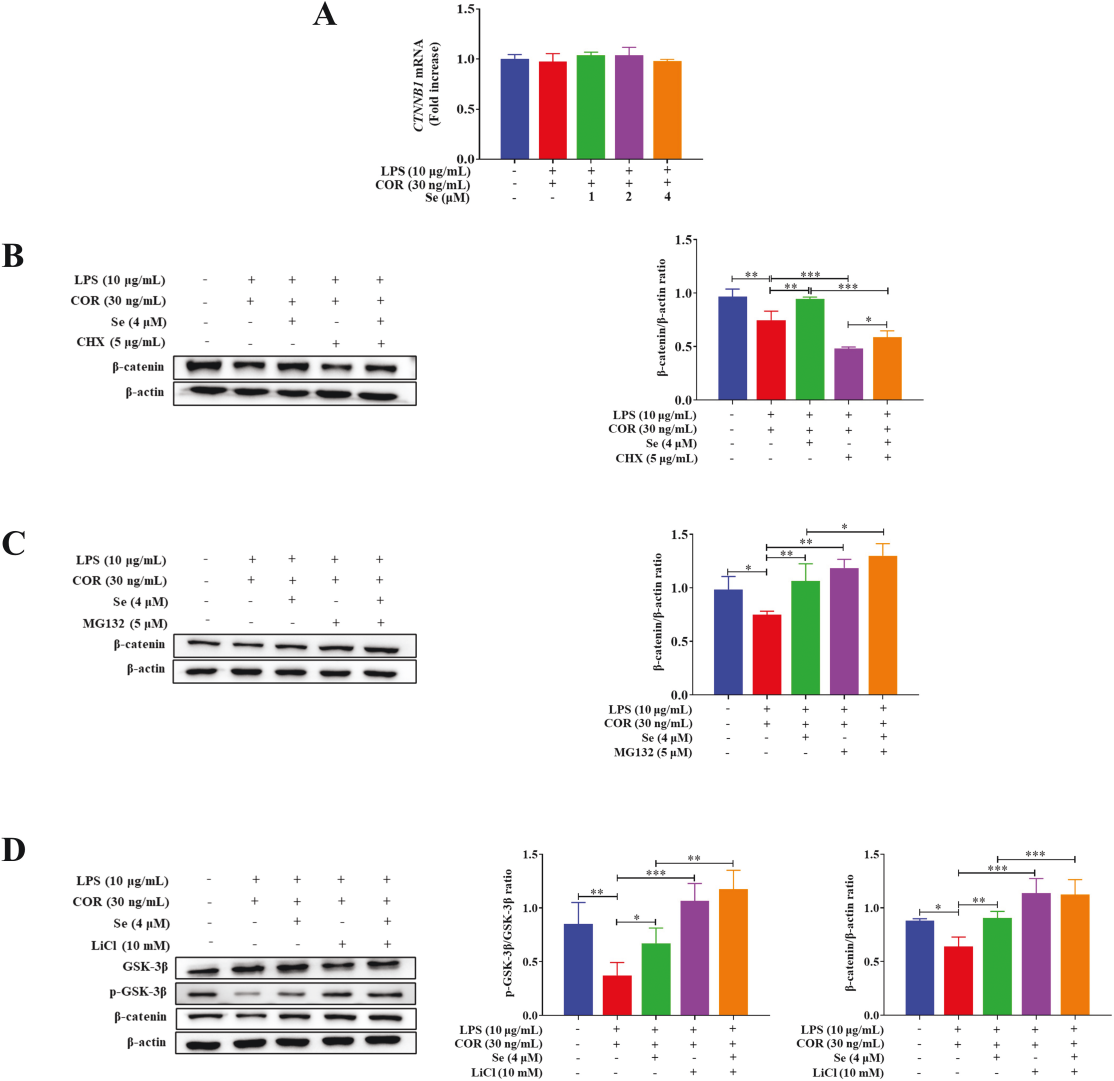
Se inhibited the LPS-induced activity of GSK-3β and accelerated the degradation of β-catenin protein through ubiquitin–proteasome pathway at high COR levels. (A) Effects of Se on the gene expressions of CTNNB1 in BEECs. The cells were co-treated with LPS and COR or co-treated with LPS, COR, and Se (1, 2, and 4 μM). (B and C) Effects of Se on the β-catenin protein levels of BEECs. (D) Effects of Se on the phosphorylation levels of GSK-3β and the protein levels of β-catenin in BEECs. (B, C, and D) The cells were co-treated with LPS and COR or co-treated with LPS, COR, and Se (4 μM) in/not in the presence of CHX (B), MG-132 (C), or LiCl (D). The phosphorylation levels of GSK-3β and the protein levels of β-catenin were detected by Western blotting. *, *P* < 0.05, **, *P* < 0.01 and ***, *P* < 0.001. All data were presented as the means ± SEM (*n* = 3).

The gene expression of *CTNNB1* was examined to ascertain whether the enhancement of the β-catenin protein was due to an altered gene transcription. The results (Figure 5A) demonstrated no apparent alteration of mRNA levels between the LPS + COR group and the LPS + COR + Se groups, indicating that β-catenin was unaffected at the transcriptional level.

Next, to determine whether the observed Se-induced up-regulation of β-catenin protein resulted from an increase in its synthesis or a decrease in its degradation, cells were treated with cycloheximide (CHX, HY-12320, MCE, USA), an inhibitor of the translation and the de novo protein synthesis (Schneider-Poetsch et al., 2010). The results (Figure 5B) showed that although CHX treatment reduced (*P* < 0.001) the β-catenin protein levels in BEECs, it was still increased (*P* < 0.05) after Se treatment in the presence of LPS, COR, and CHX. The data showed that the enhancement of β-catenin protein induced by Se probably resulted from inhibiting the degradation of β-catenin protein. To test this hypothesis, MG-132 (HY-13259, MCE), an inhibitor of the proteasome (Harhouri et al., 2017), was used in subsequent experiments. The results (Figure 5C) demonstrated that MG-132 treatment increased (*P* < 0.05 or 0.01) the β-catenin protein levels, but there was no obvious difference in the β-catenin protein levels in BEECs whether treated with Se or not in the presence of LPS, COR, and MG-132, indicating that the enhancement of the LPS-inhibited β-catenin protein levels by Se at high COR levels was the result of decreased degradation.

To further evaluate the role of the activity of GSK-3β, lithium chloride (**LiCl**, L9650, Sigma), an inhibitor of GSK-3β (Vertino et al., 2005), was utilized in additional experiments. As depicted in Figure 5D, LiCl treatment increased (*P* < 0.001) the phosphorylation levels of GSK-3β and the protein levels of β-catenin in BEECs. In addition, there was no obvious difference in the protein levels of β-catenin in BEECs whether treated with Se or not after inhibiting the activity of GSK-3β. The data showed that Se could decrease the LPS-induced degradation of β-catenin protein by inhibiting the activity of GSK-3β at high COR levels.

### Se promoted the LPS-inhibited proliferation of BEECs through the PI3K/AKT/GSK-3β/β-catenin signaling pathway at high COR levels

**Figure 6.**
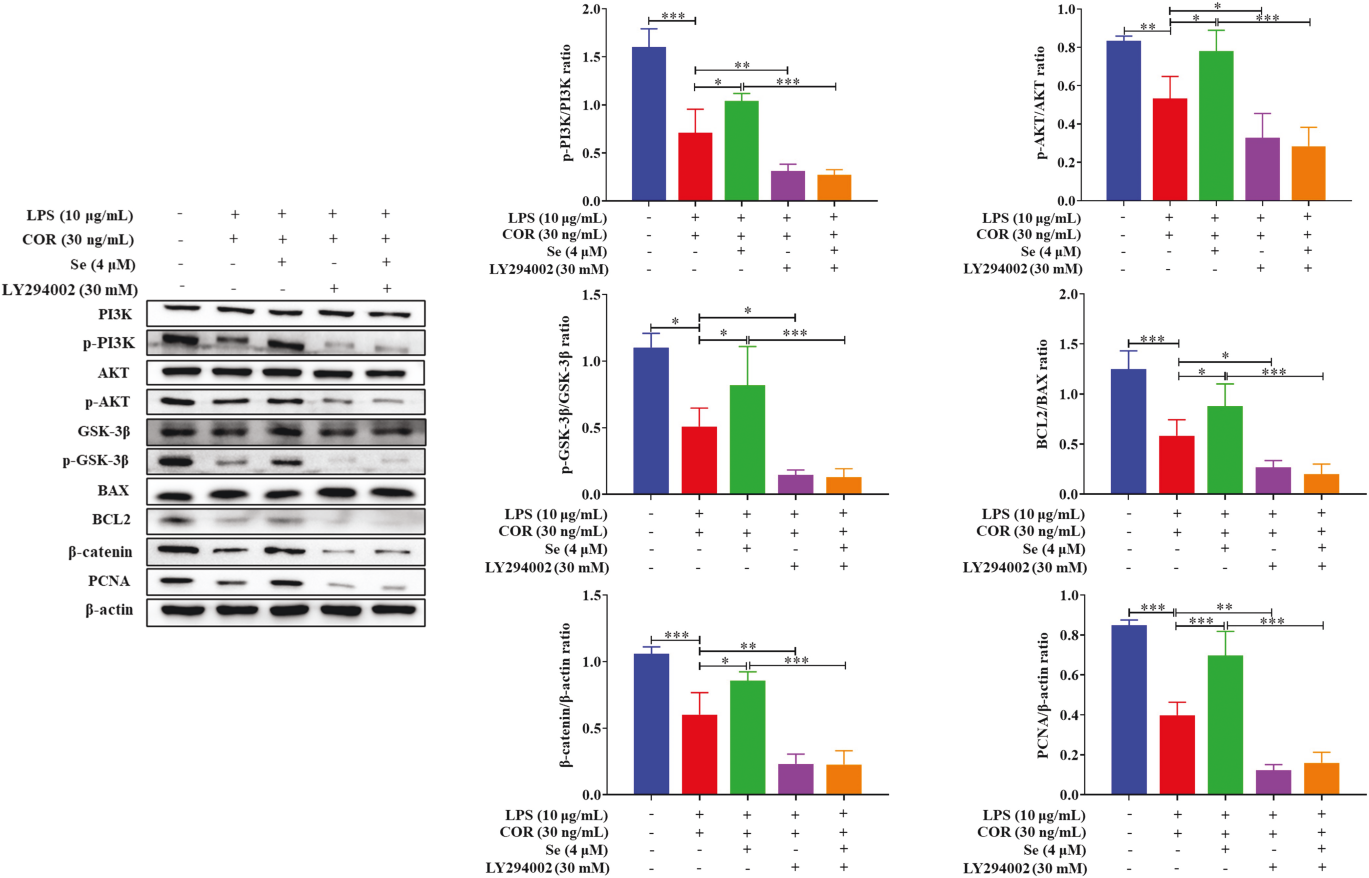
Effects of Se on the PI3K/AKT/GSK-3β/β-catenin signaling pathway. The cells were treated with co-treated with LPS and COR or co-treated with LPS, COR, and Se (4 μM) in/not in the presence of Ly294002. The phosphorylation levels the protein levels were detected by Western blotting. *, *P* < 0.05, **, *P* < 0.01, and ***, *P* < 0.001. All data were presented as the means ± SEM (*n* = 3).

**Figure 7.**
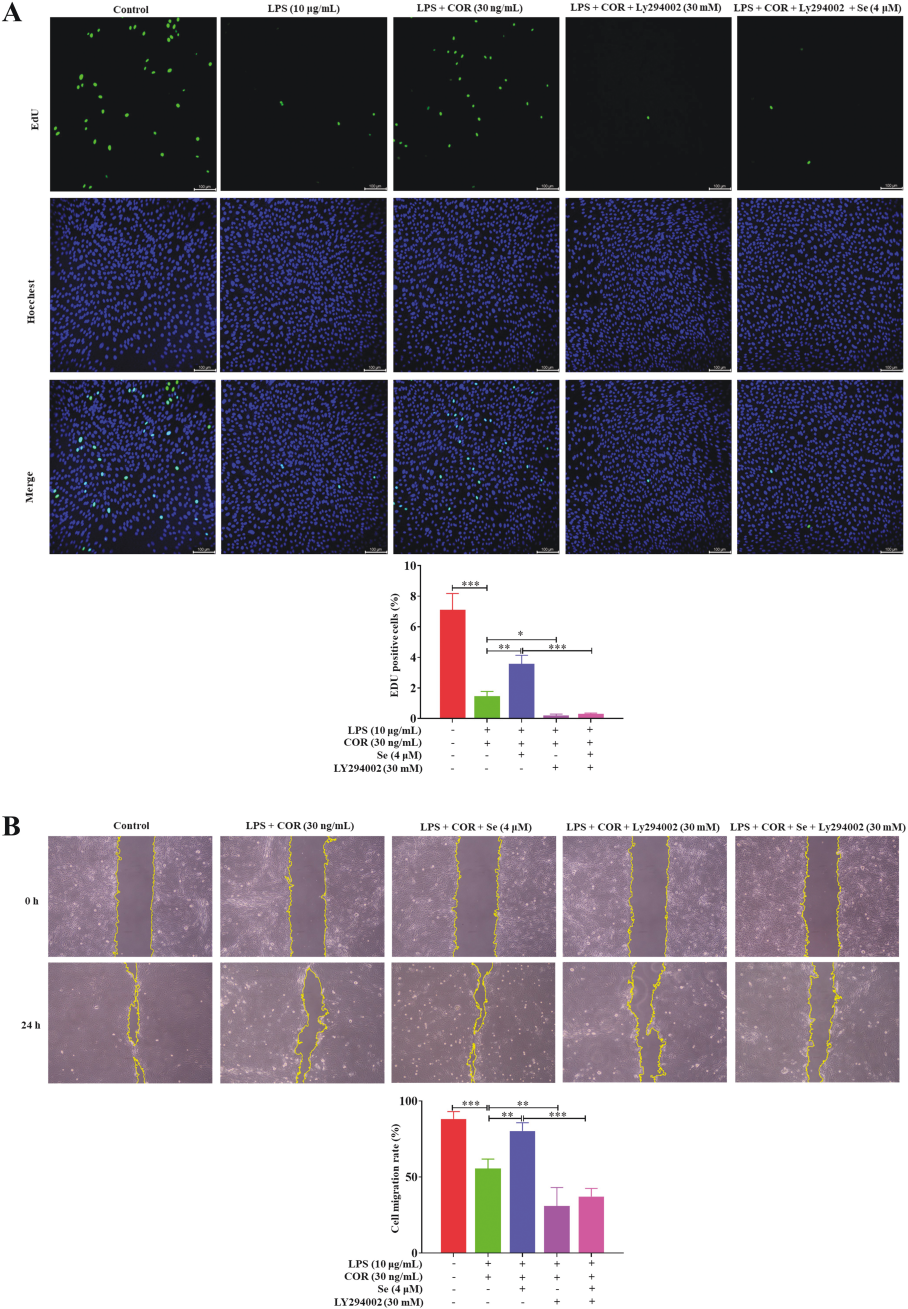
(A) The effect of Se on the cell migration rate of the BEECs by using the scratch wound healing assay. The cell migration rate = (scratch width at 0 h − scratch width at 24 h)/scratch width at 0 h × 100%. The cells were observed under light microscopy at 100× magnification. (B) EdU assay of the cell proliferation ability in BEECs. (A and B) The cells were treated with co-treated with LPS and COR or co-treated with LPS, COR, and Se (4 μM) in/not in the presence of Ly294002. *, *P* < 0.05, **, *P* < 0.01, and ***, *P* < 0.001. All data were presented as the means ± SEM (*n* = 3).

It has been well established that AKT is a known upstream kinase of GSK-3β that directly phosphorylates and inactivates GSK-3β (Cross et al., 1995), LY294002 (HY-10108, MCE), an inhibitor of PI3K (Chaussade et al., 2007), was used in experiments to further evaluate the role of the PI3K/AKT signaling pathway in the regulation of BEECs proliferation by Se. As shown in Figure 6, after inhibiting the PI3K/AKT signaling pathway with LY294002, decreases were observed in the phosphorylation levels of PI3K, AKT, and GSK-3β, the protein levels of β-catenin and PCNA, and the protein ratio of BCL2/BAX (*P* < 0.05, 0.01 or 0.001). Combined with the results of cell migration (Figure 7A) and EdU proliferation assays (Figure 7B), these findings revealed that the migration and proliferation of BEECs were inhibited (*P* < 0.05, 0.01, or 0.001). And in this state, Se supplementation did not significantly change those indexes. These results indicated that the PI3K/AKT/GSK-3β/β-catenin signaling pathway was essential for Se to regulate LPS-inhibited BEECs migration and proliferation at high COR levels.

## Discussion

Endometritis is one of the main uterine diseases that occurs in dairy cows during the puerperium period, it directly affects fertility and milk production and leads to significant economic losses (Paiano et al., 2023). A previous study has detected thirty-two strains of *E. coli* and four mixed *T. pyogenes* strains from the uteri of 19 Holstein dairy cows with obvious clinical signs. They found that virulence factor (*kpsMTII*) of *E. coli* plays a crucial role in progression of clinical metritis and endometritis (Yamamura et al., 2022). Considering the role of *E. coli* in the early stages of uterine infection, it better reflects the molecular profile of endometritis to evaluate the consequences of disease on fertility. For commercial dairies, it is essential to respond early to *E. coli* infections. Penicillin is a broad-spectrum antibiotic and is often used to prevent bacterial contamination in vitro (Seiler et al., 2016; Wang et al., 2019). Besides, penicillin was no longer added to the base medium when BEECs were treated with Se, so its effect in this experiment can be ignored. Parturition is a high-stress event with elevated cortisol. The damage to innate immune function due to the high COR levels reduces the ability of the uterus to clear bacteria and inhibits tissue repair (Zoldan et al., 2014; Alabdullah et al., 2018). Similarly, our results demonstrated that the co-treatment of LPS and COR further significantly arrested the cycle progression, reduced the secretion of related growth factors, increased cell apoptosis, and decreased cell proliferation and migration compared with the LPS group, suggesting that high levels of COR may further hinder uterine repair in cows with endometritis. Se is an essential micronutrient for mammals and is usually ingested in organic or inorganic forms through food or dietary supplements (Rayman, 2000; Carroll et al., 2015). The sodium selenite we selected in this study is clinically convenient and cost-effective. In dairy farming, it usually supplies Se in advance for a period to increase contents in cows, so we simulated the clinical situation to pre-incubate the cells. Depending on previous studies (Müller and Freude, 2016; Wang et al., 2018, 2021; Cui et al., 2023), three concentrations of Se were set to investigate the effects of Se on BEECs in the presence of LPS and COR and elucidate the related mechanism. Besides, given the clinical situation, the bovine with endometritis at high COR levels was considered as a whole, so we did not set groups treated with Se alone, Se and COR, or Se and LPS in combination.

The process of uterine repair is common to wound repair, which is dynamic and sequential. After the initial degradation of uterine tissue, bovine uterine repair is generally deemed to begin with epithelial cell regeneration (Wathes et al., 2011). As biomolecules produced by almost all cell types, cytokines play a major role in cell function (Sefat et al., 2016). Connective tissue growth factor is a member of the CCN protein family (Bradham et al., 1991). It is associated with cell adhesion, proliferation, migration, differentiation, and wound healing, and its pathological role in fibrosis has been studied extensively (Lipson et al., 2012). The activation of transforming growth factor-beta1 (**TGF-β1**) initiates a program of temporary collagen accumulation that is essential for wound repair in many organs and is involved in promoting repair of the damaged endometrium (Kim et al., 2018; Yao et al., 2019). Transforming growth factor-beta3 (**TGF-β3**) mediates a series of cellular responses, including cell survival, proliferation, migration, and differentiation (Liu et al., 2022; Du et al., 2023). Vascular endothelial growth factor (**VEGF**) is a highly conserved secreted signaling protein best known for its roles in vascular development and angiogenesis. It is emerging as an essential signaling molecule for regulating cell growth, survival, and metabolism (Shalaby et al., 1995; Wiszniak and Schwarz, 2021). These growth factors are closely linked to the proliferation of BEECs (Dong et al., 2019; Cui et al., 2021). In the present study, compared with those in the control group, the mRNA levels of these factors were significantly decreased in the LPS group, and Se treatment significantly increased the mRNA levels of TGF-β3 and VEGF in the presence of LPS and COR. Se has long been reported to promote cell proliferation and tissue repair by regulating growth factors. A previous study has found that Se probably induces angiogenesis and improves endothelial dysfunction in diabetic rats by increasing VEGF levels (Vural et al., 2017). Se-added unripe Carica papaya pulp extracts can enhance wound repair in rats through early transient expression of TGF-β1 and VEGFA at the wound area (Nafiu and Rahman, 2015). Our results suggested that TGF-β3 and VEGF may be the critical factors in the mechanism by which Se supplementation promotes uterine repair in cows with endometritis under postpartum stress, and this should be further validated in vivo.

The PI3K/AKT and Wnt/β-catenin signaling pathways are related to the proliferation of BEECs (Dong et al., 2019; Cui et al., 2021). Research has revealed that the Se and Taurine combination can promote cellular proliferation and inhibit apoptosis of bovine mammary epithelial cells through the PI3K/AKT pathway (Liu et al., 2021). Se also affects the survival of some cancer cells through the Wnt/β-catenin signaling pathway (Gong and Li, 2016; Korbut et al., 2018). The balance between anti-apoptosis protein BCL2 and pro-apoptosis protein BAX plays a key role in regulating programmed cell death in many cell types (Holohan et al., 2008). Proliferating cell nuclear antigen (**PCNA**) is an essential factor in DNA replication and repair, and it is recognized as a marker of proliferation (González-Magaña and Blanco, 2020). Besides, Cyclin D1 promotes mitosis and is pivotal for cell proliferation (Kalani et al., 2008). c-Myc controls global gene expression, regulates cell proliferation, cell differentiation, and cell cycle, and is ubiquitously expressed during tissue development (Llombart and Mansour, 2022). In the present study, Se treatment significantly activated the PI3K/AKT and Wnt/β-catenin signaling pathways, and substantially elevated their downstream the BCL2/BAX protein ratio and the protein levels of PCNA, Cyclin-D1 and c-Myc in the presence of LPS and COR. Combined with the results of the cell cycle, cell scratch, and EdU assay, Se contributed to the uterine repair by decreasing LPS-induced cell apoptosis and promoting cell proliferation through the PI3K/AKT and Wnt/β-catenin signaling pathways at high COR levels. Similarly, some studies have shown that the PI3K/AKT and Wnt/β-catenin signaling pathways are also involved in the regulation of Se on the liver health and metabolism of the broiler, and the uterine repair of goats (Li et al., 2023a, 2023b).

It has been well demonstrated that phosphorylation at the S9 site inhibits the activity of GSK-3β (McCubrey et al., 2014). However, the role of GSK-3β phosphorylation in the Wnt signaling pathway still needs to be carefully investigated. One study showed that the regulation of β-catenin by Se is GSK-3β dependent on phosphorylation in HT-29 cells but independent in HCT-8 cells (Saifo et al., 2010). In this study, we first found that β-catenin protein up-regulation was accompanied by simultaneous phosphorylation of GSK-3β at S9 down on various concentrations of Se treatment. Moreover, the PI3K/AKT signaling pathway is closely related to GSK-3β, and AKT is capable of phosphorylating GSK-3β (Rommel et al., 2001; Jope et al., 2007). Therefore, we speculated that Se may inhibit the activity of GSK-3β through the PI3K/AKT signaling pathway to regulate the Wnt/β-catenin signaling pathway to promote cell proliferation in the presence of LPS and COR. As the central signaling protein of the Wnt/β-catenin signaling pathway, the protein level of β-catenin is closely related to the expression of downstream oncogenes (Huber et al., 1996; He et al., 1998; Tetsu and McCormick, 1999). Many studies have shown that cytoplasmic β-catenin is degraded by an Axin/GSK-3β/APC complex (Behrens et al., 1998; Hart et al., 1998), and we demonstrated that the change of β-catenin protein is due to the inhibition of degradation. In human esophageal squamous cell carcinoma, Se accelerates the degradation of β-catenin to regulate cell growth (Zhang et al., 2010). Similarly, capsaicin can also affect cell migration and invasion by regulating the ubiquitination and degradation of β-catenin in human melanoma cells (Shi et al., 2021). Then, the results after LiCl treatment showed that GSK-3β was involved in the regulation of β-catenin, which is consistent with the findings of previous studies (Korbut et al., 2018). The final results after Ly294002 treatment further verified that Se promoted the LPS-inhibited proliferation of BEECs through the PI3K/AKT/GSK-3β/β-catenin signaling pathway at high COR levels. In a mouse model of Alzheimer’s disease, Se supplementation promoted hippocampal neurogenesis through the PI3K/AKT/GSK-3β/Wnt signaling pathway (Zheng et al., 2017). Selenium-enriched polysaccharides from Pyracantha fortuneana were found to inhibit β-catenin signaling in a GSK-3β-dependent mechanism to reduce the growth and invasion of human ovarian cancer cells (Sun et al., 2016). It has been reported that bone morphogenetic protein 9 induces rapid phosphorylation of GSK3-β in a mouse osteoblast cell line through the PI3K/AKT activation pathway, leading to the intracellular accumulation of β-catenin protein (Eiraku et al., 2019). Andrographolide is reported to suppress melanin synthesis in mice through the AKT/GSK-3β/β-catenin signal pathway (Zhu et al., 2015). These findings provide another molecular mechanism for the regulation of β-catenin except the Wnt signal by affecting the β-catenin degradation pathway.

The purpose of endometritis treatment is to eliminate inflammation and promote uterine repair. Moreover, one study has reported that Se could inhibit the LPS-induced inflammatory response in BEECs under high COR background (Cui et al., 2023). Goats and cows are both ruminants and share some similarities. A recent study revealed Se supplementation reduced the inflammatory response and promoted endometrial tissue repair by activating the PI3K/AKT and Wnt/β-catenin signaling pathways in goats (Li et al., 2023a). Given the ability of Se to regulate inflammation and cell proliferation, researchers have been developing selenium-containing drugs to prevent and treat disease. For example, a silk-based film containing Se nanoparticles is thought to pave the way for next-generation antimicrobial materials for applications such as wound healing and as agents against topical infections (Toprakcioglu et al., 2023). Recent studies suggest that Se can be used not only for human cancer prevention but also for treatment of cancer growth (Brozmanová et al., 2010). Selen methionine is considered to be useful in clinical treatment for renal cell carcinoma (Garje et al., 2018). Therefore, Se may be a new direction for preventing and treating bovine endometritis during the perinatal period. And the PI3K/AKT/GSK-3β/β-catenin signaling pathway we demonstrated may be an important target for research to promote uterine repair in cows. In addition, considering that this study found that a high concentration (4 μM) of Se was more conducive to improving cell proliferation than a low concentration (1 μM), we propose that appropriately increasing dietary Se concentrations may be more beneficial to bovine health and thus yield greater economic benefits.

## Conclusions

The present study demonstrated that Se supplementation could attenuate BEECs damage and promote cell proliferation and migration in the presence of LPS and high cortisol levels. This effect was possibly regulated by increasing the gene expressions of growth factors (*TGFB3* and *VEGFA*) and activating the PI3K/AKT/GSK-3β/β-catenin signaling pathway.

## Abbreviations

AKT: protein kinase B
APC: adenomatous polyposis coli
BAX: BCL2-associated X protein
BCL2: B-cell lymphoma 2
BEEC: bovine endometrial epithelial cells
CHX: cycloheximide
CK1α: casein kinase 1α
COR: cortisol
GSK-3β: glycogen synthetase kinase 3β
LEF: lymphoid enhancing factor
LiCl: lithium chloride
LPS: lipopolysaccharide
PCNA: proliferating cell nuclear antigen
PI3K: phosphatidylinositol 3-kinase
TCF: T-cell factor
TGF-β1: activation of transforming growth factor-beta1
TGF-β3: activation of transforming growth factor-beta3
VEGF: vascular endothelial growth factor
